# County-level societal predictors of COVID-19 cases and deaths changed through time in the United States: A longitudinal ecological study

**DOI:** 10.1371/journal.pgph.0001282

**Published:** 2022-11-18

**Authors:** Philip J. Bergmann, Nathan A. Ahlgren, Rosalie A. Torres Stone

**Affiliations:** 1 Department of Biology, Clark University, Worcester, MA, United States of America; 2 Department of Sociology, Clark University, Worcester, MA, United States of America; Universidad Nacional de Colombia, COLOMBIA

## Abstract

People of different racial/ethnic backgrounds, demographics, health, and socioeconomic characteristics have experienced disproportionate rates of infection and death due to COVID-19. This study tests if and how county-level rates of infection and death have changed in relation to societal county characteristics through time as the pandemic progressed. This longitudinal study sampled monthly county-level COVID-19 case and death data per 100,000 residents from April 2020 to March 2022, and studied the relationships of these variables with racial/ethnic, demographic, health, and socioeconomic characteristics for 3125 or 97.0% of U.S. counties, accounting for 96.4% of the U.S. population. The association of all county-level characteristics with COVID-19 case and death rates changed significantly through time, and showed different patterns. For example, counties with higher population proportions of Black, Native American, foreign-born non-citizen, elderly residents, households in poverty, or higher income inequality suffered disproportionately higher COVID-19 case and death rates at the beginning of the pandemic, followed by reversed, attenuated or fluctuating patterns, depending on the variable. Patterns for counties with higher White versus Black population proportions showed somewhat inverse patterns. Counties with higher female population proportions initially had lower case rates but higher death rates, and case and death rates become more coupled and fluctuated later in the pandemic. Counties with higher population densities had fluctuating case and death rates, with peaks coinciding with new variants of COVID-19. Counties with a greater proportion of university-educated residents had lower case and death rates throughout the pandemic, although the strength of this relationship fluctuated through time. This research clearly shows that how different segments of society are affected by a pandemic changes through time. Therefore, targeted policies and interventions that change as a pandemic unfolds are necessary to mitigate its disproportionate effects on vulnerable populations, particularly during the first six months of a pandemic.

## Introduction

The SARS-CoV-2 corona virus causes the disease COVID-19. It emerged in late 2019 and has spread throughout the world. As of May 1, 2022, the United States led the world in infections and mortality, with over 84 million cases and over 1 million deaths. Since the beginning of the pandemic, patterns of unequal infection and mortality have emerged across countries and communities based on factors such as behavior, race/ethnicity, demography, health, and socioeconomics, and these are often interconnected [1–5].

For example, in the U.S., Black and Hispanic communities have experienced disproportionately high COVID-19 infection and mortality rates [6–11]. Socioeconomic and health disparities resulting from structural racism have been implicated in these patterns [12–17]. The U.S. Black and Hispanic populations disproportionately reside in densely populated areas, and have lower incomes [18,19], higher unemployment, or are employed in frontline, essential occupations [4]. These communities often mistrust health care institutions [20,21], have lower access to health care due to income and lack of health insurance [22], and have a higher incidence of pre-existing conditions such as diabetes [23] than their White counterparts.

How these various factors affect rates of COVID-19 infections and death in the general population also differ. For example, high-density housing, along with frontline, essential work are likely to lead to higher rates of infection due to lack of ability to socially distance in these settings [24–26]. Meanwhile, lack of access to healthcare and higher incidence of pre-existing conditions are likely to lead to higher mortality [22,23,27]. In contrast, high unemployment may coincide with lower rates of infection due to lower exposure to other people.

County-level demographic and COVID-19 data have been valuable in understanding the disproportionate burden across communities [13,14]. However, most studies have only taken snapshots of the pandemic’s effect by using the most recent cumulative data available at the time of analysis [18,19,28]. This provides an understanding of cumulative effects since the beginning of the pandemic, but, as a result, we lack an understanding of how the pandemic has progressed through time in relation to race/ethnicity, demographic, health, and socioeconomic factors. For example, we know that Black communities show higher rates of infection and mortality [9,29], but we do not know whether the effect on these communities has worsened, remained constant, or improved through time. It is likely that the effects of the pandemic are not temporally static. In particular, the elderly comprised a high proportion of infection and mortality early in the pandemic, but as the pandemic progressed, the demographics of infection, but not mortality, shifted to younger groups [5,30–32].

Here we present an analysis of how COVID-19 infections and mortality relate to a range of racial/ethnic, demographic, health, and socioeconomic factors through time for 3125 or 97.0% of U.S. counties, accounting for 96.4% of the U.S. population. We specifically address how these relationships have changed on a monthly basis from April 2020 to March 2022. Although community-level data have important limitations, including being subject to the ecological fallacy, they provide the best means for a comprehensive understanding of patterns over time across the entire United States. Differences in data reporting across jurisdictions precludes use of more precise data that also cover the entire country [33,34].

## Materials and methods

### Variables and data sources

We conducted a longitudinal ecological study using county-level population data and sampling COVID-19 case and death data on a monthly basis. County-level demographic and health data were downloaded and compiled from the United States Census Bureau’s 2014–2018 American Community Survey for 3220 counties (www.tigerweb.geo.census.gov/tigerwebma), and from the County Health Rankings & Roadmaps (CHRR) database (www.countyhealthrankings.org). We calculated population density as the total population for each county divided by its land area, using U.S. Census data. Although we included all explanatory variables in our models, for ease of presentation, we divided them into racial/ethnic, demographic, health-related, and socioeconomic variables. We selected explanatory variables known to be predictors of COVID-19 outcomes. For racial/ethnic variables, we included the proportion of the county population that was non-Hispanic White, Black, Hispanic, Asian, and Native American. For demographic variables, we included population density, and the proportion of the population that was foreign-born non-citizen, female, and that lives in a rural setting. For health-related variables, we included the proportion of the population that was elderly, disabled, obese, and lacked health insurance. Finally, for socioeconomic variables, we included median household income, the proportion of households under the poverty line, the proportion of the population that was unemployed, or had a university degree, and the Gini coefficient, which is a measure of income inequality (zero being complete income equality, and one signifying that a single person has all the income). The specifics for each explanatory variable are listed in S1 Table. We acknowledge that many other societal characteristics and variables have been identified as related to COVID-19 cases and deaths, but wished to focus on a manageable set that is well represented in the literature.

County-level COVID-19 case and death data were compiled from The New York Times COVID-19 repository (www.github.com/nytimes/covid-19-data) [35] and normalized per 100,000 residents (we refer to these as case and death rates). We retrieved cumulative COVID-19 case and death data for the first day of each month from April 2020 until March 2022, and calculated the number of cases and deaths per 100,000 residents that occurred during each month by subtraction. These monthly case and death rates served as the response variables in our analyses. The interval that we selected coincided with the first month in which COVID-19 had spread in parts of the U.S. until the outbreak of the Omicron BA.1 variant subsided and testing was still widely available. We ended data analysis with March 2022 because as 2022 progressed, at-home testing for COVID-19 increased, often with no mechanism for patients to report test results, leading to increased underreporting of cases [36,37].

The S1 Data includes all data that we compiled and used. All data are publicly available and county-level, so their use did not require approval by the Institutional Review Board.

### Statistical analysis

We used negative binomial regression models weighted by county population to quantify the relationships between COVID-19 case and death rates, and all of the racial/ethnic, demographic, health, and socioeconomic variables that we studied. The partial slopes of these models provide estimates of relationship between each explanatory variable with the response, while taking into account all other included explanatory variables [38]. Negative binomial models are flexible in accounting for differing levels of overdispersion and zeros in the data [13,39,40], such as when many counties have no deaths during a particular month. We weighted our analyses by county population [28,41], which considerably increased the variance in COVID-19 case and death rates that our analyses explained (S2 Table).

For each month, we fitted a negative binomial regression model with the glm.nb function in the MASS package [42] using R v4.0.3 [43]. We ensured that collinearity did not compromise the analyses by calculating the tolerance of each explanatory variable. All of our variables had tolerances >0.1, which was deemed acceptable [13,38], except percent non-Hispanic White (S3 Table). To address this, we excluded this variable from our analyses, and then repeated analyses including percent non-Hispanic White and all demographic, health, and socioeconomic variables, but excluding the other racial/ethnic variables. We present results as partial slopes from the models that included all variables except percent non-Hispanic White, plus the partial slope for percent non-Hispanic White from the additional analyses. We then plotted partial slopes with their 95% confidence intervals through time. All statistical results, including p-values are presented in S4 and S5 Tables. R^2^ values for all models are presented in S2 Table. We repeated these analyses without weighting by county population (i.e., treating each county the same), and obtained mostly qualitatively similar patterns, except for the proportion of county population that lived in rural settings or were elderly (S1 Fig). We do not discuss the unweighted analyses further, but the similarity of observed patterns suggests that our results are robust to analytical choices.

## Results

**Fig 1:**
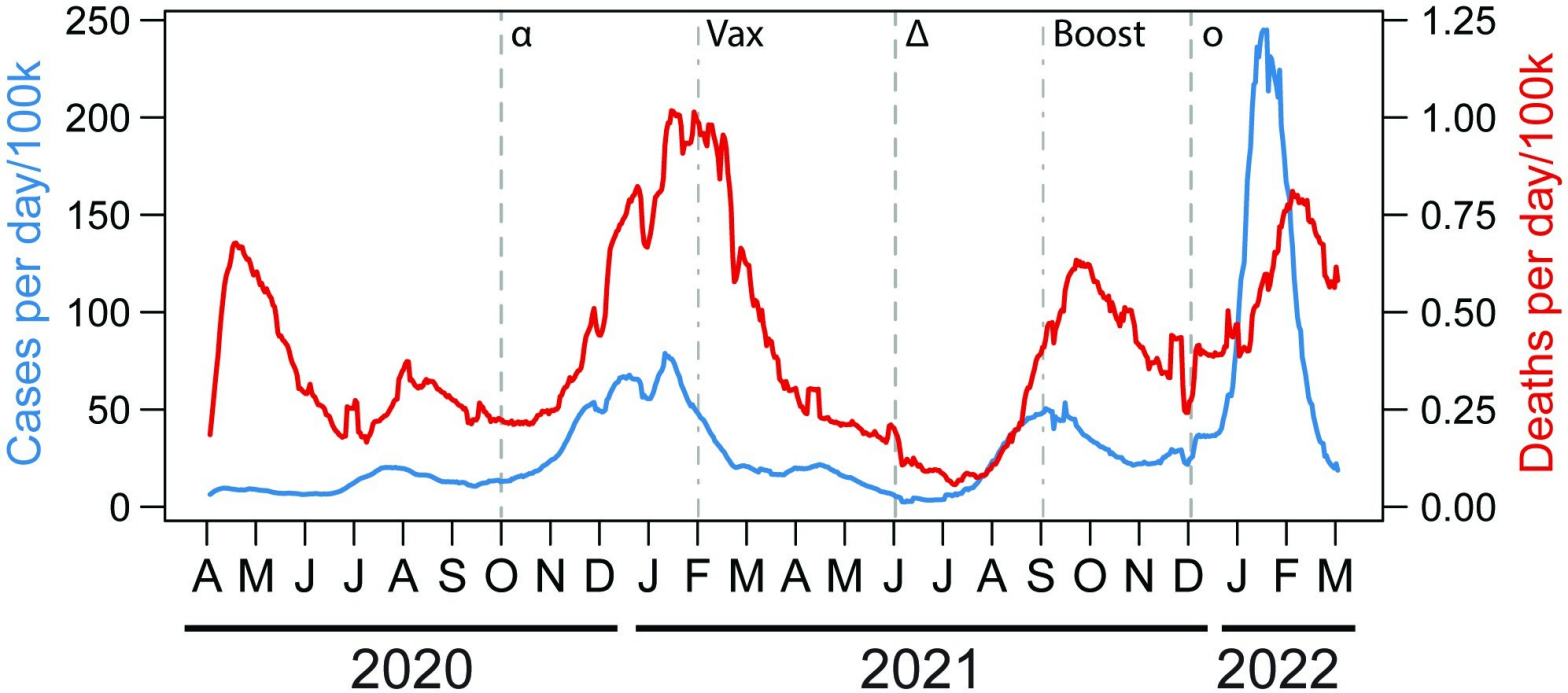
Graph of COVID-19 case and death rates for the United States through time. Vertical dashed lines indicate the approximate time when the alpha (α), delta (δ), and omicron (o) variants of SARS-CoV-2 started to spread in the U.S. Dot-dashed lines indicate when vaccination (Vax) and boosting (Boost) became widespread in the U.S. Blue lines represent case rate and red lines represent death rate.

We found dramatic changes through time in how racial/ethnic, demographic, health, and socioeconomic characteristics of U.S. counties related to per capita COVID-19 infections and deaths. The changes that we present account for all other county characteristics included in our analyses, representing their independent effects. The context for these patterns are the national COVID-19 case and death rates, which have also fluctuated (Fig 1). Additionally, a number of important events happened during the pandemic that might impact how particular groups have been affected, including the spread of the alpha (from October 2020), delta (from June 2021) and omicron (from December 2021) variants [44,45], and the widespread availability of vaccination (from February 2021) and boosters (from September 2021) (Fig 1). Over this timeframe, the models that we fit also fluctuated in the amount of variance in case (15–62%) and death (9–59%) rates that they explained (S2 Table). Many of the lower R^2^ values corresponded with months when overall COVID-19 case and death rates were low across the U.S.

### How racial/ethnic characteristics relate to COVID-19 infection and mortality

**Fig 2:**
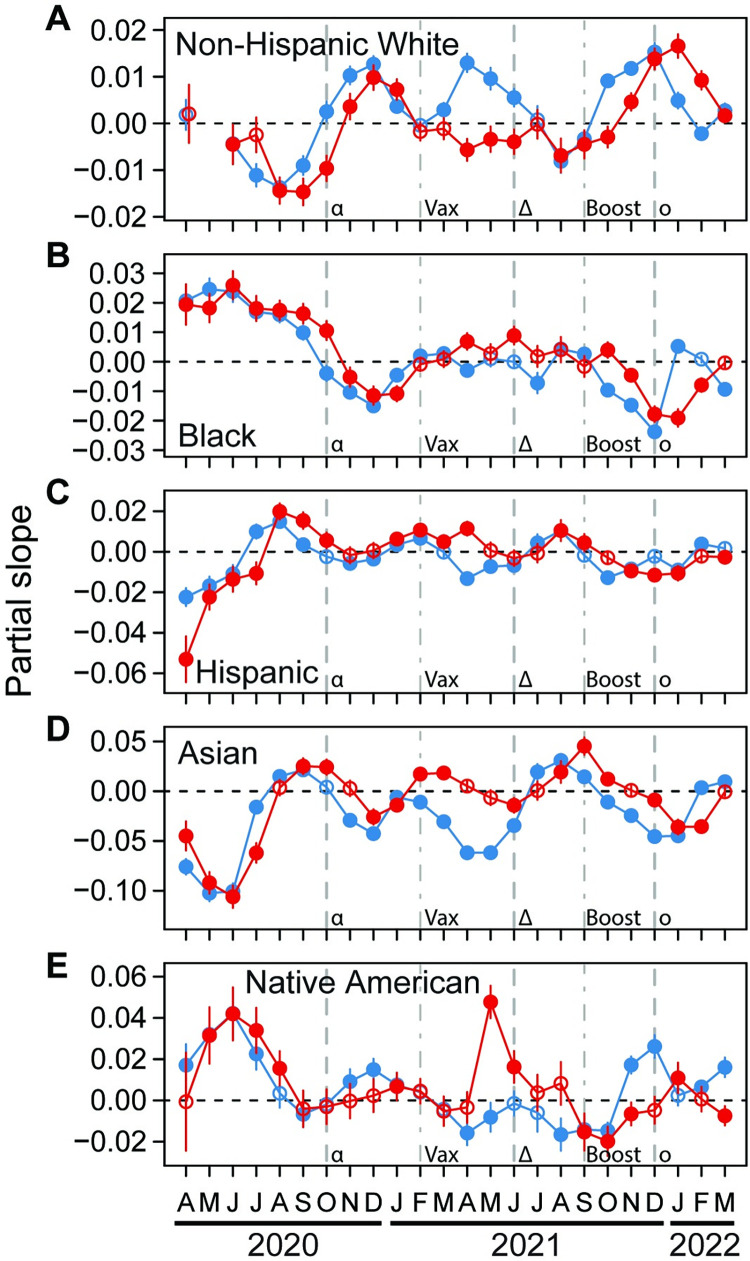
Relationships through time between either COVID-19 case (blue) or death (red) rates and proportion of U.S. county populations comprised of non-Hispanic White (A), Black (B), Hispanic (C), Asian (D), and Native American (E) residents. Partial slopes through time represent the strength of relationship for each variable. Error bars for each month are 95% confidence intervals. Filled circles are significantly different from zero, and open circles are not. Dashed lines indicate the approximate time when the alpha (α), delta (δ), and omicron (o) variants of SARS-CoV-2 started to spread in the U.S. Dot-dashed lines indicate when vaccination (Vax) and boosting (Boost) became widespread in the U.S. Models including non-Hispanic White did not converge for May 2020, resulting in missing partial slopes for that variable and that month.

The patterns of relationship between COVID-19 and the proportions of White and Black residents were approximately inverses of one another (Fig 2A and 2B). Counties with greater proportions of Black and lower proportions of White residents had higher COVID-19 case and death rates for the first seven months of the pandemic. These patterns reversed around the holidays of Thanksgiving and Christmas 2020, before disappearing round February 2021 when vaccination became widely available (Fig 2A and 2B). After that time, COVID-19 case and death rates were weakly related to proportion of Black residents, becoming negatively related towards the end of 2021, after vaccine boosters became available (Fig 2B). In contrast, after vaccination became available, counties with higher proportions of White residents tended to have higher case and death rates, but this fluctuated through time (Fig 2A). Relationships for White residence were strongly decoupled for case (positive relationship) and death (negative relationship) rates just after vaccinations became available.

We also found that counties with a higher proportion of Hispanic residents had lower case and death rates for the first four months, followed by higher case and death rates in August and September 2020, January and February 2021, and July and August 2021, with periods of weakly negative or no relationships between these dates (Fig 2C). The relationship of COVID-19 rates with proportion of residents of Asian descent were strongly negative until August 2020, followed by fluctuations between positive and negative (Fig 2D). After vaccination became widespread, relationships for case and death rates with Asian population proportion decoupled somewhat with strong negative relationships for cases and weak relationships for deaths (Fig 2D). Relationships of COVID-19 case and death rates with proportion of county residents that were Native American fluctuated through the pandemic, but were often positive, especially at the beginning of the pandemic (Fig 2E). Counties with higher population proportions of Native Americans also had much higher death rats in May and June 2021 (Fig 2E). Death rates for this group tended to be lower after vaccine boosters became available (Fig 2E).

### How demographic characteristics relate to COVID-19

**Fig 3:**
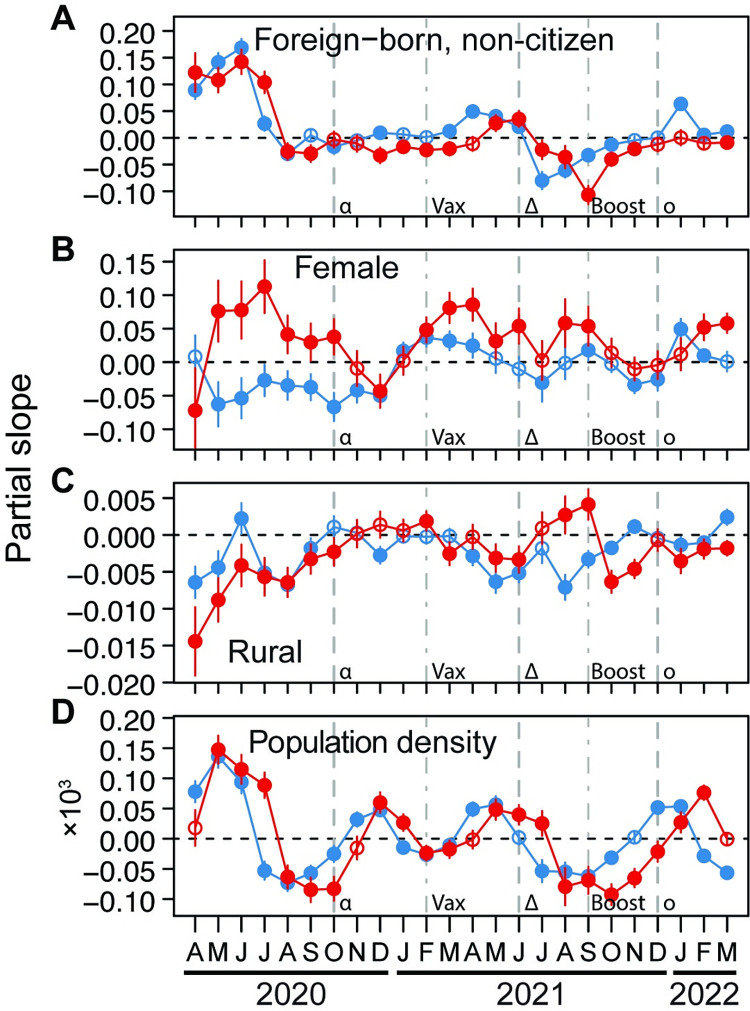
Relationships through time between either COVID-19 case (blue) or death (red) rates and proportion of U.S. county populations comprised of foreign-born non-citizens (A), females (B), and residents living in rural settings (C), as well as the county population density (D). Partial slopes through time represent the strength of relationship for each variable. Error bars for each month are 95% confidence intervals. Filled circles are significantly different from zero, and open circles are not. Dashed lines indicate the approximate time when the alpha (α), delta (δ), and omicron (o) variants of SARS-CoV-2 started to spread in the U.S. Dot-dashed lines indicate when vaccination (Vax) and boosting (Boost) became widespread in the U.S.

The relationships of COVID-19 case and death rates with the proportion of county residents that were foreign-born non-citizens were closely coupled and strongly positively related during April to July 2020 (Fig 3A). From August 2020 until February 2021, the relationships between COVID-19 and this demographic were either not different from zero or slightly negative. Counties with higher foreign-born non-citizen populations had lower case and death rates in July to October 2021, coinciding with the spread of the delta variant, before relationships became weak or non-existent again (Fig 3A). Counties with higher proportion of female residents had lower case rates but higher death rates from May until October 2020, followed by fluctuating patterns that were more coupled between case and death rates, and mostly positive (Fig 3B). The proportion of county population living in a rural setting was strongly negatively related to both case and death rates for the first six months of the pandemic, followed by weakly negative relationships (Fig 3C). However, in August and September 2021, more rural counties had higher death rates (Fig 3C). Finally, county population density was strongly positively related to case and death rates during the first few months of the pandemic, then negatively related in August to October 2020, followed by fluctuations between positive and negative relationships (Fig 3D). The relationship between population density and case and death rates was negative while the delta variant dominated in the U.S., and then became more positive when the omicron variant spread (Fig 3D).

### How health-related characteristics relate to COVID-19

**Fig 4:**
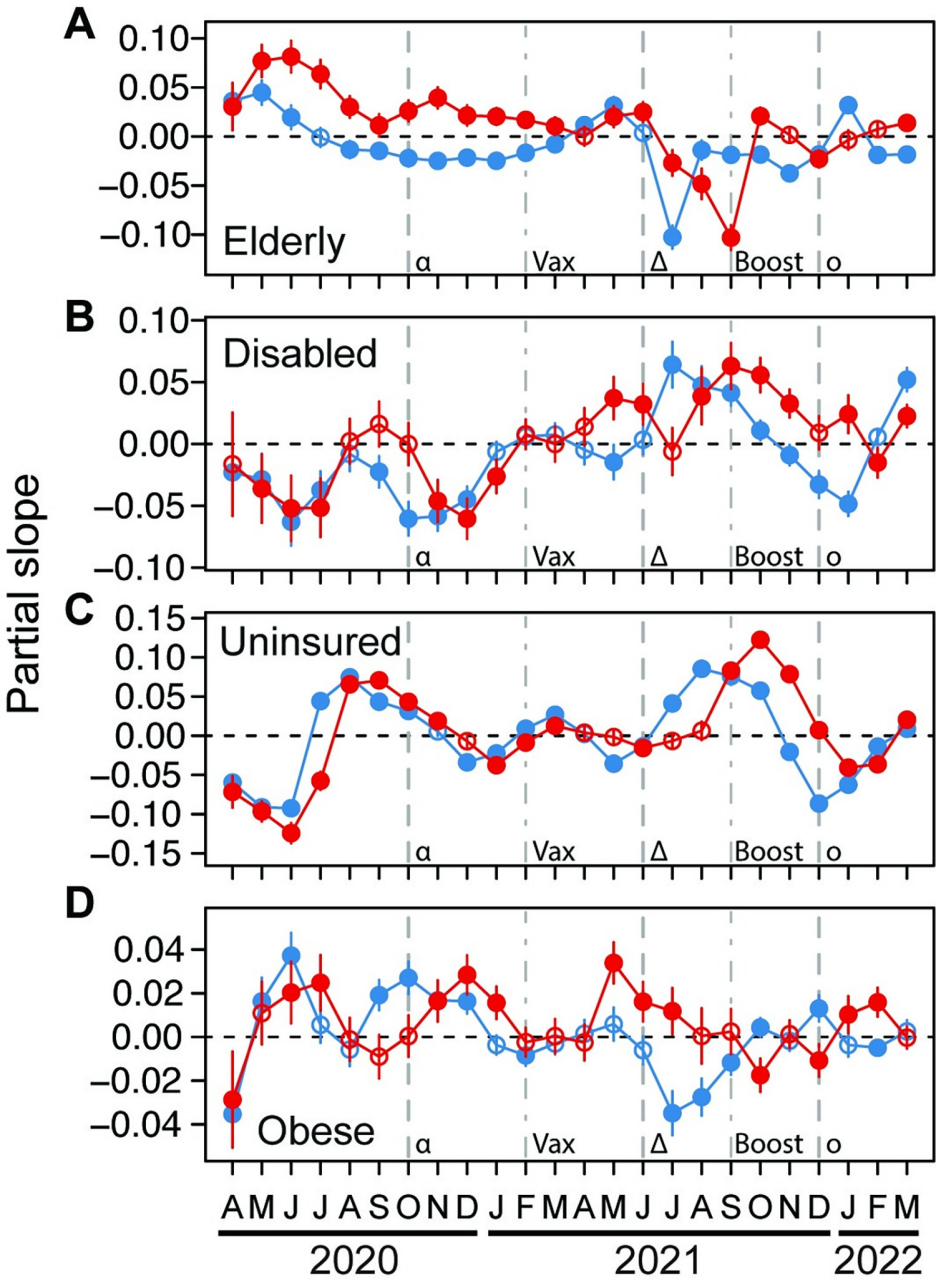
Relationships through time between either COVID-19 case (blue) or death (red) rates and proportion of U.S. county populations comprised of elderly (A), disabled (B), health uninsured (C), and obese (D) residents. Partial slopes through time represent the strength of relationship for each variable. Error bars for each month are 95% confidence intervals. Filled circles are significantly different from zero, and open circles are not. Dashed lines indicate the approximate time when the alpha (α), delta (δ), and omicron (o) variants of SARS-CoV-2 started to spread in the U.S. Dot-dashed lines indicate when vaccination (Vax) and boosting (Boost) became widespread in the U.S.

Counties with a higher population proportion that was elderly had higher COVID-19 case rates for the first three months of the pandemic and higher death rates for the first year (Fig 4A). For most of the pandemic, case rates were negatively related to elderly population proportion. Death rates were positively related until they became strongly negatively related in July to September 2021, and then remained weak after boosters were widespread (Fig 4A).

The proportion of county population that was disabled or lacked health insurance fluctuated with case and death rates in similar ways (Fig 4B and 4C). The relationships to death rates lagged slightly in time relative to case rates for both variables. There was a negative relationship between COVID-19 and both of these variables for the first four months of the pandemic, followed by fluctuations that became stronger from about July 2021, when the delta variant spread (Fig 4B and 4C). Specifically, proportions of disabled and uninsured residents were positively related to COVID-19 case and death rates from about July to about November 2021, followed by weaker or negative relationships afterward (Fig 4B and 4C).

The relationship between proportion of county population that was obese with both COVID-19 case and death rates was strongly negative in April 2020, then quicly becoming positive and fluctuating until about April 2021, when relationships to case and death rates became more decoupled (Fig 4D). From May to September 2021, counties with more obese populations had higher death rates but lower or unrelated case rates. After September 2021, case and death rates fluctuated weakly between positive and negative (Fig 4D).

### How socioeconomic characteristics relate to COVID-19

**Fig 5:**
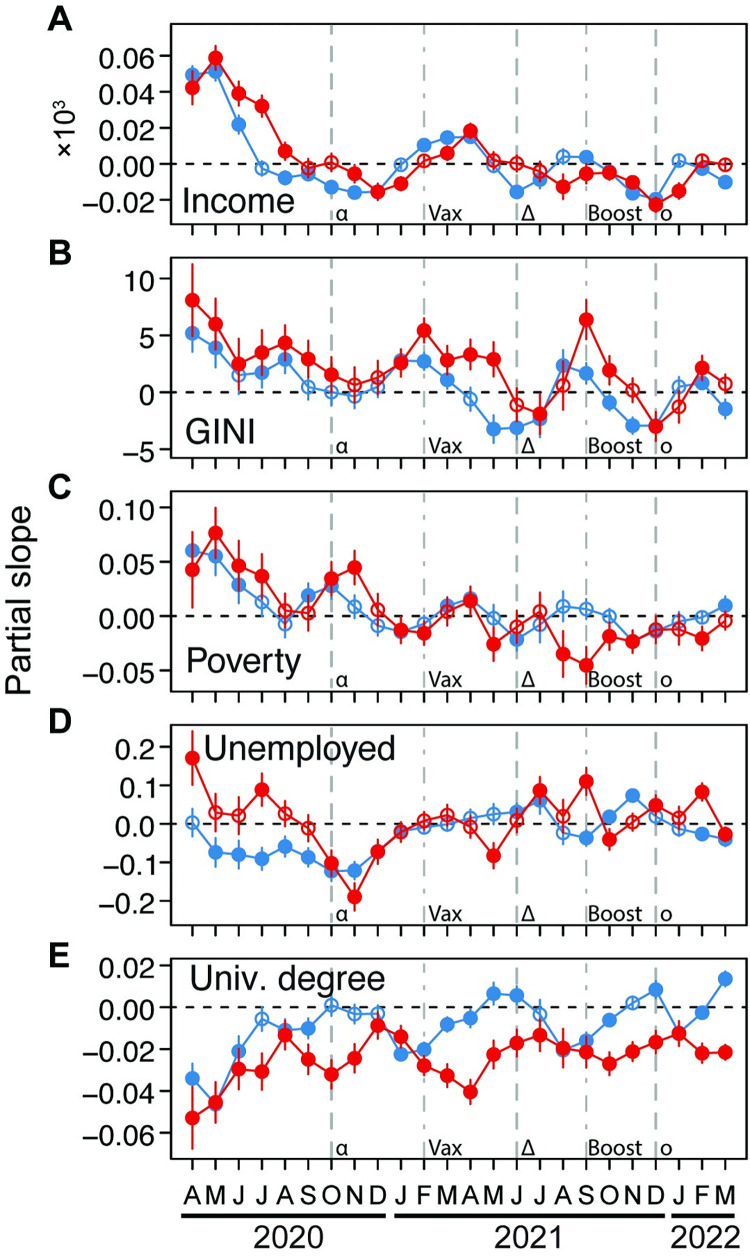
Relationships through time between either COVID-19 case (blue) or death (red) rates and U.S. county median income (A), proportion of households below the poverty line (B), proportion of residents that unemployed (C), and proportion of residents with a university degree (D). Partial slopes through time represent the strength of relationship for each variable. Error bars for each month are 95% confidence intervals. Filled circles are significantly different from zero, and open circles are not. Dashed lines indicate the approximate time when the alpha (α), delta (δ), and omicron (o) variants of SARS-CoV-2 started to spread in the U.S. Dot-dashed lines indicate when vaccination (Vax) and boosting (Boost) became widespread in the U.S.

Median county income, the Gini index (a measure of income inequality), and the proportion of households in poverty had similar temporal relationships with COVID-19 case and death rates (Fig 5A, 5B and 5C). For the first four to six months of the pandemic, counties with higher incomes, income inequality, and higher poverty had higher case and death rates. Subsequently, all three variable fluctuated through time in their relationship with COVID-19, with fluctuations become less pronounced and lower for median income and poverty (Fig 5A and 5C), while being more sizeable for income inequality (Fig 5B). From July 2021 until March 2022 (the end of our sampling), counties with higher incomes and poverty had either no relationship with COVID-19 case or death rates, or negative relationships. In contrast, counties with higher income inequality had higher case and death rates January to May, as well as August to September 2021, with intervening periods of negative relationship or no relationship (Fig 5B).

The proportion of county populations that were unemployed had negative relationships with COVID-19 case rates, and positive or not significant relationships with death rates through September 2020 (Fig 5D). Subsequently, the relationship between unemployment and death rate became mostly negative and coupled with patterns for case rates. From about July 2021 until March 2022, case and death rates fluctuated between being positively and negatively related to unemployment rates (Fig 5D).

Finally, the proportion of county populations with a university degree was the most consistent predictor of COVID-19 case and death rates. Throughout much of the pandemic, counties with higher proportions of university-educated residents had lower death rates and mostly lower case rates (Fig 5E). However, in May, June and December 2021, as well as March 2022 counties with more highly educated populations had higher case rates, although death rates remained lower (Fig 5E).

## Discussion

In our study, we tested whether and how the relationships between COVID-19 and a range of racial/ethnic, demographic, health, and socioeconomic factors changed through time across the U.S. Most importantly, we found that not only do these relationships change through time, but they differ between COVID-19 case and death rates, and between societal factors at the county level. Our findings provide an important step in understanding how a pandemic affects different segments of society as it progresses, and has important implications, particularly that policies and practice for mitigating the effects of a pandemic must also change through time.

That some racial and ethnic groups have been disproportionately affected by COVID-19 is well established [13,16,26,27,46], but how this has changed temporally was not well understood. We found that counties with a higher proportion of the population that is Black had higher case and death rates for the first six months of the pandemic, before this pattern reversed, and then fluctuated through time (Fig 1B). This shift closely coincided with the holidays of Thanksgiving, Christmas, and the New Year of 2020, and repeated for 2021, when travel across the country peaked despite warnings from public health officials [1,47]. It seems probable that widespread travel indiscriminately spread COVID-19, and that this served to either obfuscate racial disparities observed at the beginning of the pandemic, or reflect racial differences in holiday travel. That the relationships between COVID-19 with proportions of White and Black individuals were somewhat inverse in temporal pattern supports this (Fig 1A and 1B). However, this inverse pattern must be interpreted cautiously because the proportion White population variable was highly collinear with all other racial categories (S3 Table). On the other hand, the inverse pattern was only observed between Black and White groups, not other racial groups. Nevertheless, the strong effect of COVID-19 on counties with high proportions of Black residents were likely due to the additive effects of systemic racism [12,24,48], but appear to have been mitigated to some degree as the pandemic progressed. The disproportionate impact on U.S. Black populations is even more striking given a higher biological susceptibility to COVID-19 of populations of European descent than those of African descent due to a genomic segment, inherited from Neanderthals, that is prevalent in the former and virtually absent in the latter [49,50]. However, there are also regional differences in these patterns in the U.S. [15,51,52] that we did not consider here.

In contrast, the temporal patterns of how county proportions of Hispanic, Asian, and Native American residents did not change around the year-end holidays, but had strong patterns especially at the beginning of the pandemic. There are important cultural and socioeconomic differences within both the Hispanic and Asian populations, so ethnic subgroups within these categories may be differentially affected by the pandemic [53–55]. Counties with higher population proportions of foreign-born individuals that were not U.S. citizens also had higher case and death rates early in the pandemic, but subsequently, this demographic was related to mildly lower case and death rates (Fig 2A).

The patterns that we documented in how median income, income inequality (Gini index), and household poverty were related to COVID-19 case and death rates were similar (Fig 5A–5C). In particular, counties with higher incomes, income inequality, and poverty suffered higher COVID-19 case and death rates for the first seven months of the pandemic, followed by fluctuating relationships. A positive relationship between income inequality and COVID-19 has been documented internationally and in the U.S. and is a proxy for socioeconomic disadvantage [56–58]. Similarly, poor households also tend to be more crowded with smaller living spaces [59], and poverty corresponds with lower access to healthcare in the U.S. [29], likely leading to worse outcomes. The pandemic also produced disproportionate job loss and food and medical insecurity among low-wage subpopulations [4]. The matching temporal pattern between median income, income inequality, and poverty is counterintuitive, but may be due to cost of living differences across counties [60].

The results discussed above showed that the first six months of the pandemic had many of our explanatory variables strongly related to COVID-19 case and death rates. After this time, relationships changed, likely because society responded more effectively to the pandemic. For example, during that initial stage of the pandemic, counties with higher population proportions of Black, Native American, foreign-born, elderly, and obese residents suffered higher case and death rates. Additionally, counties with higher density populations, high income inequality, and high poverty rates suffered higher case and death rates, and these counties also tended to have higher median incomes. These patterns reveal that segments of society were particularly vulnerable to the pandemic when it began, but also suggest some success of society in responding to these vulnerabilities, as relationships became less pronounced after that period.

The contrasting temporal patterns of how population density and proportion of the population living in rural settings related to COVID-19 infections and mortality (Fig 3C and 3D) suggest that the independent effects of these variables measure different things. That population density related strongly and positively to mortality, both at the beginning of the pandemic and after the alpha and omicron variants became widespread, may correspond with factors such as ability to isolate effectively when infected in high-density settings. It also may represent differences between urban, low-density housing such as the suburbs versus urban high-density housing [18].

We found evidence of human behavior affecting COVID-19 progression for some segments of the population. For example, except for the first three months of the pandemic, case rates were largely either unrelated to the elderly county population, or negatively related (Fig 4A). It is likely that elderly people took considerable precautions, such as mask wearing and social distancing [61] to avoid infection and that public health practices improved as the pandemic progressed. Similarly, mortality in counties with higher elderly populations improved after vaccination became widespread (Fig 4A), consistent with the observed high uptake of vaccines by this demographic [62]. However, older age remains an important predictor of COVID-19 outcomes [32,63,64]. Counties with a greater proportion of the population with a university degree consistently had lower COVID-19 case and death rates (Fig 5E), with behavior again being the most likely explanation. In particular, people with a university degree were more likely to be employed in situations where they could work from home to facilitate social distancing [65], and more likely to accept vaccination [62].

The observed weak relationships between unemployed residents and COVID-19 case and death rates (Fig 5D) may be rationalized in that unemployed people had few workplace interactions, and likely engaged in less travel for work, recreation, and shopping [66]. During the first six months of the pandemic, proportion of county populations that were disabled was also either unrelated or weakly negatively related to COVID-19 case and death rates (Fig 4B). Similarly, this may be explained in that persons with disabilities may have lower mobility, resulting in lower potential for exposure and infection [29]. Subsequently, relationships between COVID-19 case/death rates fluctuated dramatically and similarly with population proportions of disabled and residents that lacked health insurance (Fig 4B and 4C). These similar patterns are harder to explain. However, the strongest positive relationships between these groups and COVID-19 coincided with the autumn 2021 peaks in COVID-19 case and death rates (Fig 1), and this suggests an inability of these groups to avoid COVID-19 during at least that particular surge [67].

### Limitations

County-level data have important limitations, including being subject to the ecological fallacy, but provide the best means for a comprehensive understanding of temporal patterns across the entire U.S. County-level data are an amalgamation of populations, limiting the ability to make definitive conclusions about mechanisms. However, differences in data reporting across jurisdictions precludes use of more precise data that also cover the entire country [33,34]. Some of the county-level social variables (S1 Table: percentage of the population that lacks health insurance, is obese, or is unemployed) that we used are modeled, and data for counties with low populations or low response rates to surveys in particular are estimates based on those models [68]. Therefore, these estimates may be less accurate than direct data for the other variables we included [68], which are based directly on data. These estimated variables may be collinear with other variables partly as a result of being estimated using other variables.

Another limitation is differential COVID-19 data quality across jurisdictions. U.S. states have maintained different data reporting standards, including for counting cases and deaths, and the frequency of reporting. Differences in COVID-19 testing availability exacerbated this. Therefore, deaths and especially cases are undercounted [69]. How this might bias the results is unknown and likely complex. Weighting our analyses by county population should address some of these biases by increasing the weight of counties with higher populations and more resources for counting cases and deaths. Weighted analyses explained more variance in COVID-19 case and death rates than unweighted analyses (S2 Table). Finally, we note that the explanatory power of our models changes substantially through time, with less variation in COVID-19 case rates being explained between March and July 2021, and June to August 2021 for death rates (S2 Table). This period of time mostly coincided with low case and death rates nationally (Fig 1), which may explain the low explanatory power.

Given that the COVID-19 case and death data are temporal in nature, a time series analysis is another option for analysis. We did not use a time series analysis because COVID-19 is an emerging and quickly evolving disease and monthly sampling for only two years (24 time points) is insufficient for a robust analysis [70,71]. This might be remedied with a shorter time interval (e.g., weekly), but then the number of cases and particularly deaths would be zero for most counties during most time points, also weakening the analysis.

## Conclusions

We showed that relationships between racial/ethnic, demographic, health, and socioeconomic factors with COVID-19 case and death rates changed through time in the U.S. Temporal changes and differences in how particular population segments are infected and die from COVID-19 are critical to informing policy and practice behind mitigation efforts, especially in resource-limited scenarios such as a pandemic. This could include prioritizing efforts to mitigate spread versus enhancing access to health care. For example, we found that counties with higher Black, Native American, foreign-born, elderly, high density, and impoverished populations were particularly susceptible to infection and mortality from COVID-19 early in the pandemic. Efforts to address factors leading to the spread of the virus and higher mortality of these vulnerable groups are particularly important at the onset of a pandemic. A health equity lens to mitigate COVID-19 disparities is key. For example, enhancing access to testing in places where these groups are more likely to receive care [72], adopting more racially equitable triage in racially diverse areas [73], and addressing implicit biases in medical treatment [74] would all help address these disparities. The first six months of a pandemic appear to be critical in addressing these issues, and so learning from the COVID-19 pandemic should be applied to any future pandemics. Additionally, the consistently negative relationship between university education and COVID-19 case and death rates highlights a well-established positive impact of education on health outcomes and mitigating health disparities [75]. Finally, our results suggest that efforts to decrease the spread of COVID-19 among more elderly populations were somewhat successful, especially later in the pandemic, but efforts to decrease mortality in this demographic took much longer to establish.

## Supporting information

S1 Fig Temporal results for unweighted analyses. (PDF)

S1 Table List and descriptions of explanatory variables. (XLSX)

S2 Table R2 values for weighted and unweighted models. (XLSX)

S3 Table Tolerances for explanatory variables used in models. (XLSX)

S4 Table Negative binomial model results for analyses including all explanatory variables except non-Hispanic White. (XLSX)

S5 Table Generalized linear model results for analyses including non-Hispanic White. (XLSX)

S1 Data Publicly available data used in the analyses. (XLSX)
