# Supplementary material for: County-level societal predictors of COVID-19 cases and deaths changed through time in the United States: A longitudinal ecological study
Source: PLOS Glob Public Health. 2022 Nov 18;2(11):e0001282. doi: 10.1371/journal.pgph.0001282 (PMC10022229; doi:10.1371/journal.pgph.0001282)
Supplement: S1 Fig — (PDF) [file pgph.0001282.s001.pdf]

S1 Figure for "County-level societal predictors of COVID-19 cases and deaths changed through time in the United States: A longitudinal ecological study"

Philip J. Bergmann, Ph.D.*[1], Nathan A. Ahlgren, Ph.D.[1], Rosalie A. Torres Stone, Ph.D.[2]

Affiliations:
[1]Department of Biology, Clark University, Worcester, MA, 01610, USA
[2]Department of Sociology, Clark University, Worcester, MA 01610, USA
*Corresponding author: pbergmann@clarku.edu

S1 Figure
Temporal results for analyses not weighted by county populations.
Relationships through time between either COVID-19 case (blue) or death (red) rates and each explanatory variable for U.S counties. Partial slopes through time represent the strength of relationship for each variable. Error bars for each month are 95% confidence intervals. Relationships are significant if error bars do not cross the zero line. Dashed lines indicate the approximate time when the alpha (α), delta (δ), and omicron (o) variants of SARS-CoV-2 started to spread in the U.S. Dot-dashed lines indicate when vaccination (Vax) and boosting (Boost) became widespread in the U.S. Explanatory variables are listed in each graph.

S1 Figure

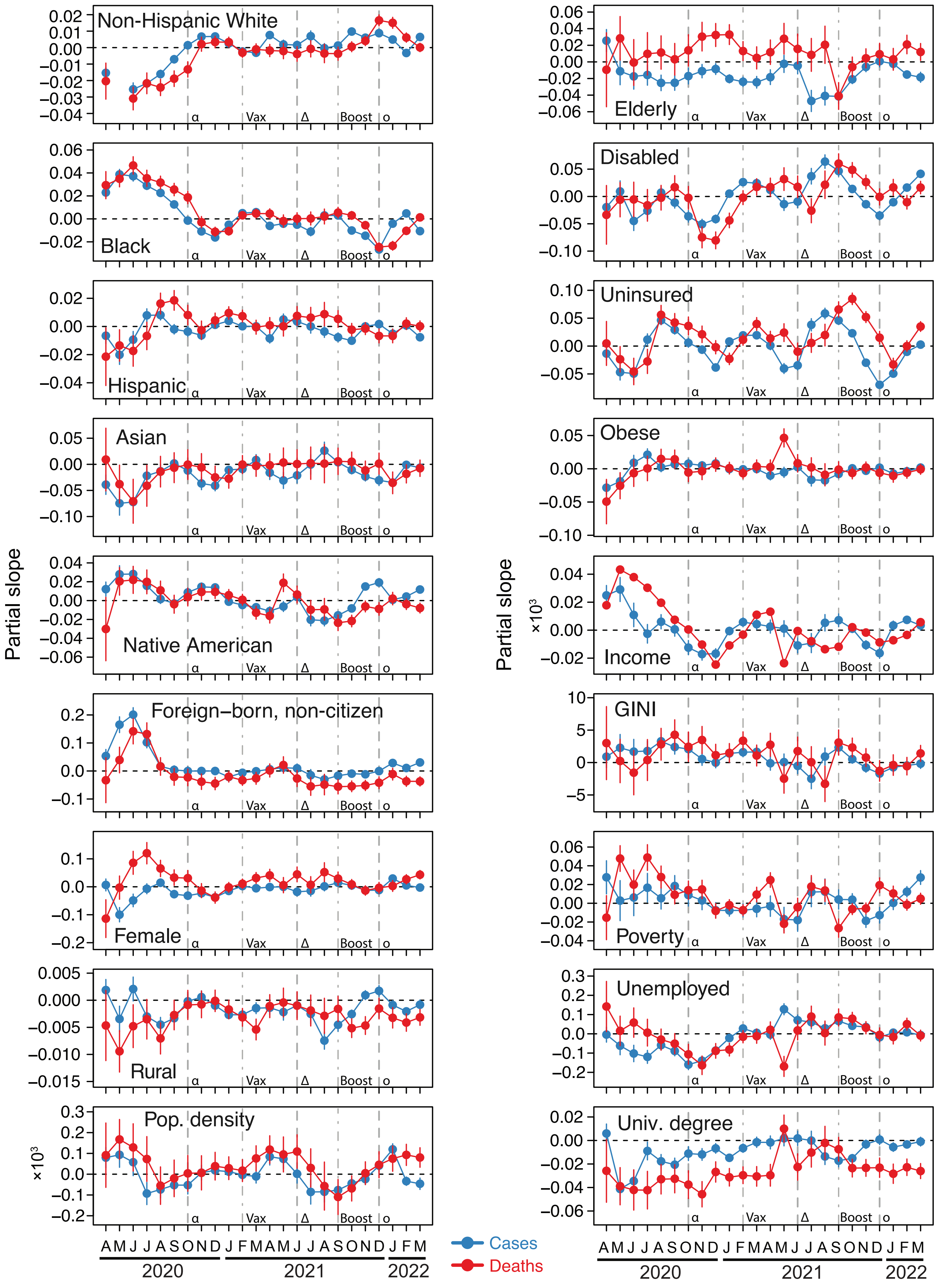
Non-Hispanic White
Black
Hispanic
Asian
Native American
Foreign–born, non-citizen
Female
Rural
Pop. density
Elderly
Disabled
Uninsured
Obese
Income
GINI
Poverty
Unemployed
Univ. degree
Partial slope
×10³
α
Vax
Δ
Boost
o
A M J J A S O N D J F M A M J J A S O N D J F M
2020
2021
2022
Cases
Deaths
